# Application of the Intervention Mapping Framework to Develop an Integrated Twenty-first Century Core Curriculum—Part Three: Curriculum Implementation and Evaluation

**DOI:** 10.3389/fpubh.2017.00285

**Published:** 2017-11-01

**Authors:** Jaime A. Corvin, Rita DeBate, Kate Wolfe-Quintero, Donna J. Petersen

**Affiliations:** ^1^Department of Global Health, College of Public Health, University of South Florida, Tampa, FL, United States; ^2^Department of Health Policy and Management, College of Public Health, University of South Florida, Tampa, FL, United States; ^3^University of South Florida, Tampa, FL, United States

**Keywords:** public health, Masters in Public Health foundational core, competencies, experiential learning, pedagogy

## Abstract

Public health professionals have been challenged to radically reform public health training to meet evolving demands of twenty-first century public health. Such a transformation requires a systems thinking approach with an interdisciplinary focus on problem solving, leadership, management and teamwork, technology and information, budgeting and finance, and communication. This article presents processes for implementing and evaluating a revised public health curriculum and outlines lessons learned from this initiative. To date, more than 200 students have participated in the initial pilot testing of this program. A rigorous process and outcome evaluation plan was developed and employed. Results from the evaluation were used to enhance the resulting curriculum. Specifically, all instructional materials were evaluated by both the students who received the materials and the faculty who presented the materials. As each successive pilot is delivered, both enrollment and faculty involvement has increased. Through this process, the value of committed faculty, the importance of engaging learners in the evaluation of an education program, and the need to implement curriculum that has been carefully evaluated and evidence-informed in nature has emerged. We credit our successful transformation of the Masters in Public Health core to the challenge provided by the Framing the Future task force, the commitment of our College of Public Health leadership, the engagement of our faculty, and the time we allowed for the process to unfold. Ultimately, we believe this transformed curriculum will result in better trained public health professionals, interdisciplinary practitioners who can see public health challenges in new and different ways.

This article is *Part three* of a three-part series of articles published in *Frontiers of Public Health*. This article is preceded by:

Part 1: Application of the Intervention Mapping Framework to Develop an Integrated 21st Century Core Curriculum: Mobilizing the Community to Revise the MPH Core Competencies (1).
Part 2: Application of the Intervention Mapping Framework to Develop an Integrated 21st Century Core Curriculum: Translation of MPH Core Competencies into an Integrated Theory-based Core Curriculum (2).

## Background and Rationale

The Lancet Commission Report (2010) called for radical reform in the training of health professionals to meet the evolving demands of twenty-first century public health. This report, coupled with calls from public health professionals nationally, challenged the fragmented and outdated curriculum of the past, arguing for a more integrated, flexible, and forward-thinking curriculum designed to better prepare students for the dynamic and changing global public health environment (3, 4).

While it can be argued that all medical- and health-related curricula requires transformation, public health education, in particular the Masters in Public Health (MPH) degree, provides the perfect platform for this transformation. The issues plaguing health globally are multicausal and require integrated, interdisciplinary approaches to create sustainable and effective solutions. An antiquated, traditional, and siloed approach to foundational public health courses falls short in adequately addressing these issues. While an understanding of the traditional content (i.e., epidemiology, biostatistics, health policy and management, environmental and occupational health, and social and behavioral sciences) is critical, curricula focusing on twenty-first century public health requires a broader approach to understanding health challenges and necessitates practice-based approaches. Simply, a twenty-first century curriculum requires a system thinking approach with an interdisciplinary focus on problem solving, leadership, management and teamwork, technology, and information, budgeting and finance, and communication. This curriculum must be global in nature and reflect health across the life course. Students should be equipped with practical experience from analytical skills to writing, and emphasis should be placed on lifelong learning, relationship building, and collaboration. Further, this curriculum should be based on strong evidence and thoroughly evaluated. To fulfill this challenge, the College of Public Health (COPH) at the University of South Florida (USF) engaged in a multiyear, systematic process using the Intervention Mapping (IM) framework (5) to transform our MPH education into an integrative, comprehensive, and skills-based program to better meet the needs of the twenty-first century learner, while ensuring a stronger practitioner armed with the tools required to combat emerging public health challenges. A major accomplishment of the transformation process was the development of ten interdisciplinary MPH core competencies that respond to the field’s demand that academicians make a conscious effort to rethink public health education, as well as our own desire for a stronger, more rigorous, efficient and effective public health degree program (3, 6). These revised MPH core competencies provided a rigorous integration of traditional core curricular content within an expanded scope that includes systems thinking, globalization and sustainability, informatics, leadership and management, program planning, advocacy, and communication (blinded for review, 2017). Such curricular goals are designed to meet the new realities of professional education in public health in the twenty-first century.

In addition, rigorous educational guiding principles were designed, informing how the COPH proceeded in developing the curriculum for this foundational core, as well as how all courses and programs will be revised in the future (blinded for review, 2017). Specifically, preliminary work by the Transforming the MPH (TMPH) team at the USF determined that public health education in the twenty-first century needed to be clearly differentiated from the BSPH and the DrPH degree programs and designed in a rigorous, competency and skill-based fashion, while being grounded in applied public health practice and inclusive of global health perspectives and content. To ensure the development of a strong foundation for the public health learner’s education and career growth, the content had to be delivered to students in their first year by an interdisciplinary team of faculty and delivered as a series of integrated learning experiences rather than as a set of distinct core disciplines. Through this new curriculum, the concepts from the traditional public health disciplines that are related to health systems, determinants of health, and health assessment tools were utilized for public health skills development in an integrated fashion. In addition, critical thinking, ethics, leadership, writing, communication, and data analysis are embedded throughout the proposed content, using experiential learning techniques to help guide the effective uptake of the information (blinded for review, 2017).

Vital to this process is the successful piloting, implementation, and evaluation of this program. At the cornerstone of public health is the focus on developing evidence based programs and initiatives, grounded in theory and evaluation. Yet, it can be argued that this same approach does not translate into the classroom, and education in general is often criticized for its lack of evidence-informed implementation (7). This case study attempts to help fill this gap by describing how one university employed the IM framework (5) to develop a theory- and evidence-based integrated public health core curriculum based on the content- and skills-based knowledge required for twenty-first century public health practice. Additionally, the authors drew upon effective principles of health education and program evaluation, adopting a public health approach for the implementation and evaluation of the resulting program (8). This article presents processes for implementing and evaluating a revised public health curriculum and lessons learned from this initiative.

## Competencies and Standards Underlying the Activity

The MPH degree at the USF COPH is accredited by the Council on Education in Public Health (CEPH) and, thus, the core curriculum at USF must meet the CEPH Foundational Core Competencies. To complete this task, a committee of individuals, known as the TMPH committee was challenged to transform the curriculum in preparation for the changing CEPH competencies. This process is outlined in detail elsewhere (blinded for review, 2017). The resulting curriculum, elaborated in this article, was designed and implemented to ensure a rigorous educational platform through which all graduates of the program are grounded in foundational public health knowledge with a focus on the profession and science of public health and the factors related to human health. The program meets CEPH’s 22 MPH Foundational Competencies and the following domains: (a) evidence based approaches to public health, (b) public health and health care systems, (c) planning and management to promote health, (d) policy in public health, (e) leadership, (f) communication, (g) interprofessional practice, and (h) systems thinking.

## The Learning Environment

As the first school of public health in the State of Florida, The COPH at the USF has always been a leader in public health education and innovation. Thus, being the first school in the state, and among the first nation-wide to transform our core education was paramount. Fully accredited by the Council on Education for Public Health (CEPH), the COPH has awarded 4,235 masters degrees through the Fall of 2016. With an average graduate cohort of over 800 students, the COPH is committed to providing the highest quality, rigorous public health education to our students. Our student body is diverse; 72% are women, 46% are underrepresented minorities, 76% are Florida residents, and 10% are international students.

## Implementing a Revised Pedagogical Framework

### Intervention Mapping

The IM framework (5) was employed to guide the development of an integrated MPH core curriculum at the USF. In this article, we focus on the process and outcomes of IM steps 5–6. Step 5 focuses on curriculum implementation, while the sixth and final step includes the piloting, revision, and evaluation of the curriculum. Each step is outlined below.

### IM Step 5: Curriculum Implementation

Step 5 focuses on ensuring a strong foundation for the implementation and sustainability of the new curricula by planning how the teaching and learning plans are implemented and sustained given the instructors (i.e., implementers) and the students (i.e., adopters). To meet this challenge, it was necessary to identify individuals who could be drivers of change—those faculty who would be instrumental in the development and sustainability of this new transformed curricula—the implementers. Further, while it is necessary to start with a core of committed faculty for development, it is then equally necessary to broaden participation so that other faculty can gain understanding and contribute to the new approach. Thus, the TMPH curriculum committee included one or two faculty members from each department plus a faculty member whose specialty is writing and communication, all of whom were committed to the transformation of the MPH core.

Faculty involved in this process was given .15FTE development time in the semester before the course. Inclusive in this time was also faculty training, as all participating faculty received a series of trainings and attended sessions on various teaching modalities, including the flipped class approach. During the implementation phase, the courses were team-taught and all faculty received between .10FTE and .25FTE. The number of course sessions taught and the associated assignments were used to calculate this FTE, ensuring that faculty had adequate out-of-class time for grading the extensive writing and critical thinking assignments. Evaluation determined that approximately 10 h per session was required for preparation and grading time.

It was also necessary to identify those students who, as early adopters, would provide critical insight into the adaptation of this new material. To achieve this aim, the TMPH committee developed recruitment materials to solicit pilot volunteers from the incoming MPH class of 2014, and provided an information session at orientation to address students’ concerns and questions. This committee also submitted curriculum changes that allowed students to participate in either the traditional core or the new transformed core and earn credit toward their degree. To ensure success, it was decided that the pilot would be delivered the first time to on-campus, full-time students who self-selected the option. It was stressed to students that while this approach was innovative and involved an interdisciplinary team of instructors, which was a clear benefit, it was in the initial testing phase and might appear disorganized at times.

**Figure 1:**
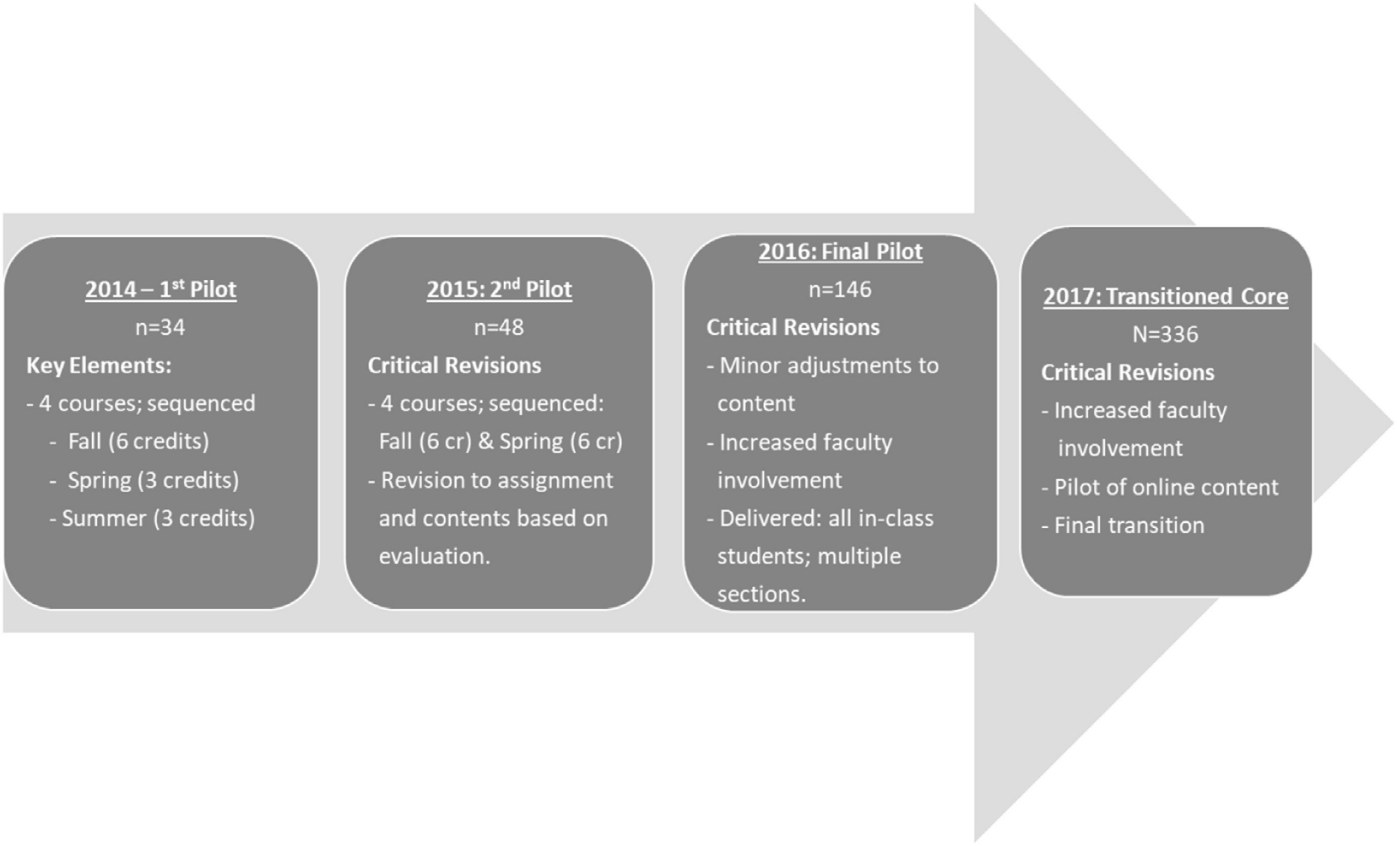
Transitioning the Masters in Public Health core.

A group of 34 students participated and completed the first pilot. In 2015, a second pilot was delivered to 48 students, also self-selected. In 2016, a final pilot was delivered to all on-campus students, with multiple sections delivered to 146 students. Finally, in 2017, the transformed core will become the official core at USF and will be required of all in-class and online students (Figure 1).

### IM Step 6: Evaluation Plan

A rigorous evaluation plan was developed to enhance both the process and outcomes. Specifically, all instructional materials were evaluated by both the students who received the materials and the faculty who presented the materials. Through the process evaluation, students provided anonymous evaluative feedback for each activity, reading, and presentation. All lesson materials were evaluated for acceptability immediately after delivery and again at the end of the semester. Students were asked guided questions about the usefulness of the material, ease of use, how the material informed understanding, and how they would rate the overall material. Students were also asked questions regarding their perceptions of how the material related to the competencies and objectives. In addition, students were asked to elaborate on what they liked about each session and what they would change. Students provided extremely constructive feedback about each element of the course. The results of the weekly evaluations were reviewed in real time by a team of faculty focused on evaluation. In some cases, real time changes could be made to instantly help clarify instructions or content. For example, emergent issues with timing or lack of understanding were immediately corrected. In these instances, the instructors immediately made revisions. In addition, the ratings for each item, along with acceptability, ease of use and understanding were carefully evaluated. Content with lower scores were flagged for follow-up in live debriefing sessions.

In addition to the anonymous evaluation, live debriefing sessions were held for each course at the end of each semester. During these sessions, students were given the opportunity to discuss their perceptions of the course. This also allowed time for the faculty to bring divergent responses in the anonymous evaluations to the entire group for feedback and additional clarification. As an example, in many cases a single lesson was noted as having both “too much” and “not enough” reading from participants. In the live session, students were asked to elaborate on this. During these sessions, students shared their opinions and perceptions about the scope of work. These conversations were incredibly helpful in allowing the evaluators to better understand the meaning behind the initial data. In the case with the reading, we were able to elucidate that simply many students didn’t want to read and this criticism did not reflect sound practice in graduate education. In this same scenario, other students made a strong case for needing more reading. Therefore, while the majority of students may have reported that the reading was “too much,” a determination was made to actually increase the amount and types of readings that students are exposed to, while implementers also became aware of the need to add incentives (e.g., quizzes) for completing the assignments. In every case, the evaluators and implementers used both sets of data to make determinations moving forward.

A stakeholder evaluation of the entire curriculum was also done by a team of faculty and students after the first pilot had been delivered, based on the process evaluation results. Based on triangulation of feedback, changes were made to the curriculum prior to piloting a second time. These revisions were particularly focused on changes to the sequencing of the course material for better flow and ease of understanding. For each successive pilot delivery, the process evaluation continued to be implemented.

Finally, to assess outcomes, performance on the Certified in Public Health (CPH) exam, generally taken in the second year of the MPH, is being compared between students who took the transformed MPH core and students who took the traditional MPH core. While results are still preliminary, to date, students in the transformed MPH core have a significantly higher pass rate (88%) when compared to students who took the exam during the same period (October 2015 to May 2017) in the USF traditional core (71%) or the national average (70%). Additional analysis will be conducted as more of the TMPH students complete the CPH exam.

## Program Implications and Lessons Learned

### Program Implications

**Figure 2:**
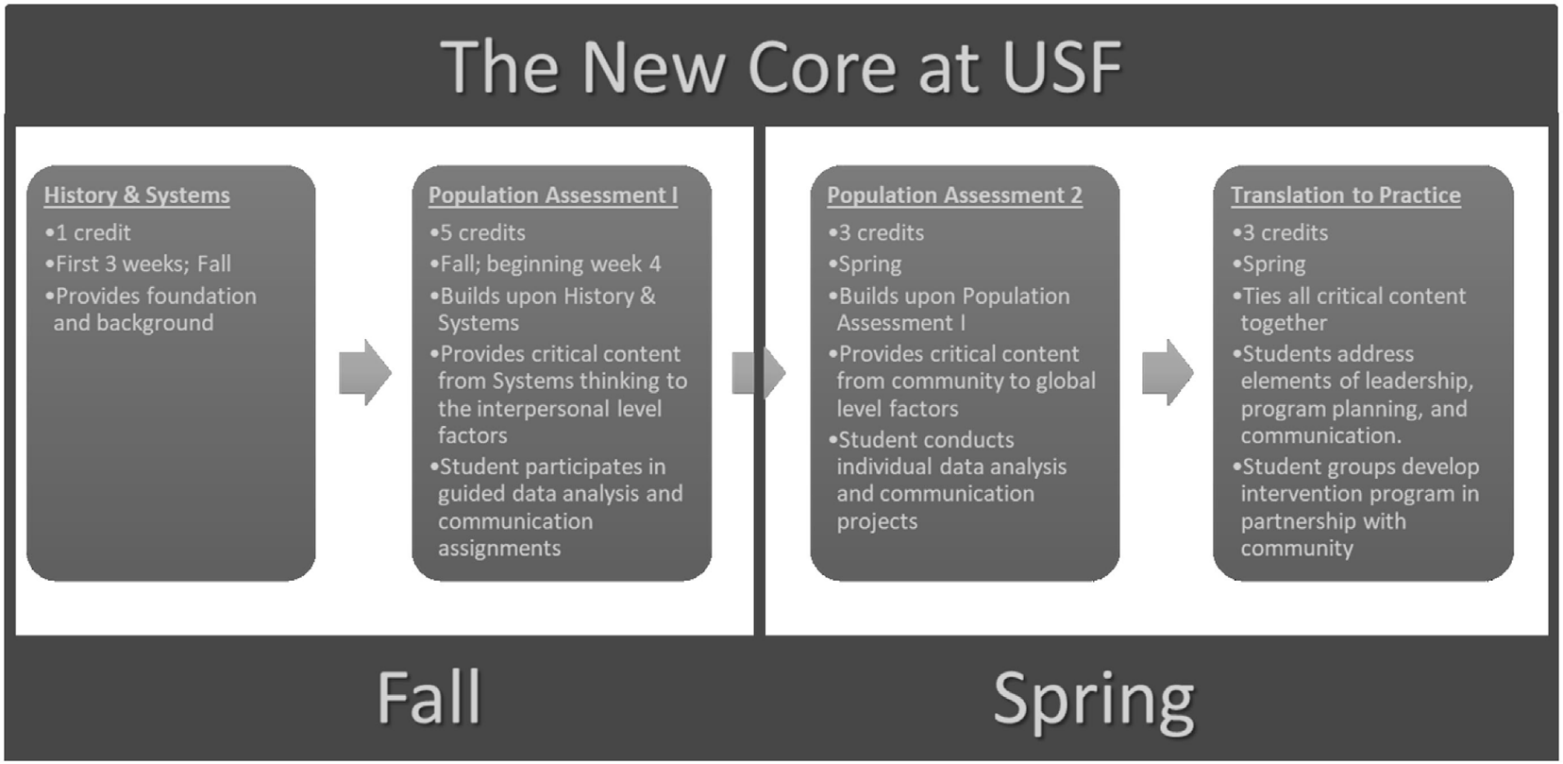
Course sequencing in the transformed Masters in Public Health core.

Masters in Public Health students are expected to take the new foundational courses as a cohort in their first year of the degree. Figure 2 illustrates this new core curriculum and how students progress through the program. This transformed core is offered for a total of 12 credits, instead of the 15 credits utilized for the traditional core. Because of the interdisciplinary integration achieved in the transformed core, we were able to remove the redundancies revealed in the traditional core, add critical twenty-first century content, and increase our expectation that students would operate as public health professionals, developing and applying public health skills with rigor, from the very start of the MPH program. Such revisions have created opportunity for concentrations to dive deeper into public health content and provide additional specialized course offerings for student.

The transformed core also allows us to orient students to successful completion of the CPH exam earlier in the program, while allowing us to eliminate our Capstone course, which was designed to integrate the disciplines and prepare students for the CPH exam; both goals are now accomplished in the transformed core. As a result of the transformed core, a total of six credits were removed from the MPH degree foundational courses, leaving room for other opportunities, such as more experiences in the field, or advanced practice electives in more than one discipline. We believe the final model addressed all of the concerns that emerged from the analysis of the data done by the TMPH team and ultimately resulted in a stronger program design.

### Lessons Learned

As a result of engaging in this process, critical lessons were learned. Specifically, public health curriculum requires a multiyear faculty effort. This effort should include a team who conceives the plan and initiates the transformation. Once planned, it is imperative to include a wider range of faculty to develop the initial teaching materials. Finally, to ensure sustainability and ownership, it is imperative that a large number of faculty are included in the efforts to deliver the curriculum, particularly as it moves from pilot to core curriculum with increasingly larger student groups, both on-campus and online. It is imperative to note that this also requires acknowledging fairly the amount of work involved and rewarding faculty accordingly. Faculty who initially resisted this process are now far more accepting once they have seen the outcomes, and many more are getting involved in curriculum delivery as we move forward.

Our process also revealed the importance of feedback from the primary stakeholder, our students. Development of successful lessons requires process feedback at the lesson level, at the module level, at the course level, in a large class focus group to gain interpretations of students’ earlier responses, and with a small stakeholder group of highly motivated students and faculty who work together to develop a new iteration of the curriculum. The information gathered through each of these processes were invaluable and helped to better inform the curriculum. It is also important to note that in each subsequent iteration of the pilot, students who were successful early adopters were hired as implementers (TAs) for the next group of students. These individuals have become agents of change and leaders in our college. They work tirelessly to help the faculty to enhance the curriculum based on evaluation data, while also working directly with the next cohort of students to ensure understanding and competence. And from them, we can see the hope for the future of public health education. This team of students sees no boundaries in public health. They are “public health practitioners” in the truest sense. Each of them has a strong grasp on data analysis, understands the importance of heath policy, thinks about ethics, is concerned about health disparities, and think about issues from the biological to the global in culturally competent ways. Never being trained in a silo allows them to help faculty think about the content in new and different ways as well.

Finally, this process supports the use of IM, designed for development and delivery of public health interventions, as an appropriate model for public health curriculum design. IM guides the process from needs assessment, through goals, objectives, delivery methods, and lessons, to completing a process evaluation. This encourages a systematic process for making critical decisions at every point of development.

## Discussion

The goal of the transformation process was to develop an interdisciplinary MPH core curriculum based on twenty-first century public health skill needs. Several iterations of curriculum teams have worked together on this goal. In the Fall of 2014, the first team of implementers began delivering a pilot version of the transformed curriculum. This pilot is ongoing and will not be completed until we have a version available for our online students in Fall of 2017. Until then, some of our students have continued to take the traditional core, leaving the college in a transitional stage.

As each successive pilot is delivered (once per year), we have increased the participation of both students and of the faculty throughout the college. Inclusion of additional faculty has been paramount as this helps to encourage ownership and commitment to the new curriculum. We have also begun evaluating the educational outcomes, by having students in each pilot rate each lesson delivered, by creating a team of students from each successive cohort to participate in a process of evaluating the curriculum, and by tracking comparative outcomes on the CPH exam as students approach graduation. It is a long inclusive process to develop and deliver a fully transformed and integrated core curriculum, but we are well on the way.

While an outcome evaluation is ongoing and the initial sample is biased based on self-selection, preliminary data suggest that students participating in the transformed core are better prepared for the CPH exam, with a significantly higher pass rate when compared to students in our traditional core. Anecdotally, we have also begun to hear from faculty that students are entering their courses with a better understanding of public health overall. Students are now prepared to manage and analyze data, write detailed public health reports, and understand the linkages between complex content. Additionally, because of the practical and hands-on nature of the courses, students better understand the real world applicability of the work they do. Throughout the courses, students are already working with and in the community, they have seen first-hand the effects of inequities, they can put a face and a name to health disparities, and they have looked both within and globally to contemplate public health crises. As the first pilot of students graduate and enter the workforce, we are also beginning to hear from students themselves about how well the program prepared them for the practice of public health. Our next steps are to conduct a formal outcome evaluation, which will include a in-depth analysis of CPH exam results, student attainment and postgraduation employment.

## Conclusion

The transformation process is a long and difficult one. The next implementation goal in this process is to develop online delivery of these materials for 2017. At this time, all incoming MPH students will be required to enroll in the transformed MPH core. Throughout this process, the team-teaching model will continue, with an ever-increasing number of faculty involved in refining and delivering each module of the curriculum. Additionally, as a thorough process and outcome evaluation is conducted, additional enhancements will likely be made to this curriculum, with the goal of ensuring a strong, evidence-informed public health curriculum for meeting the needs of the twenty-first century and beyond.

## Author Contributions

All of the authors of this article contributed in the development and design of the reported curricula, as well as in the development, writing, and editing of this manuscript.

## Conflict of Interest Statement

The authors declare that the research was conducted in the absence of any commercial or financial relationships that could be construed as a potential conflict of interest.
